# Non-Mycobacteria Tuberculosis in Africa: A Literature Review

**DOI:** 10.4314/ejhs.v33i5.21

**Published:** 2023-09

**Authors:** OT Ojo, AO Odeyemi

**Affiliations:** 1 Department of Medicine, Lagos State University Teaching Hospital, Ikeja, Lagos, Nigeria; 2 Department of Medicine, College of Medicine, Lagos State University, Lagos, Nigeria; 3 Department of Medicine, College of Health Sciences, Osun state University, Osogbo, Nigeria; 4 Department of Internal Medicine, UNIOSUN Teaching Hospital, Osogbo, Nigeria

**Keywords:** Atypical mycobacteria, Tuberculosis, Non-Tuberculous mycobacteria in Africa

## Abstract

**Background:**

Non-tuberculous mycobacteria (NTM) have been reported to cause pulmonary and extrapulmonary infections. These NTMs are often misdiagnosed as MTB due to their similar clinical presentations to tuberculosis, leading to inappropriate treatment and increased morbidity and mortality rates. This literature review aims to provide an overview of the prevalence, clinical manifestations, diagnosis, and management of NTM infections in Africa.

**Methods:**

A systematic search was performed using various electronic databases including PubMed, Scopus, and Web of Science. The search was limited to studies published in the English language from 2000 to 2021. The following keywords were used: “non-tuberculous mycobacteria”, “NTM”, “Africa”, and “prevalence”. Studies that focused solely on the Mycobacterium tuberculosis complex or those that did not report prevalence rates were excluded. Data extraction was performed on eligible studies. Overall, a total of 32 studies met the inclusion criteria and were included in this review.

**Results:**

In our literature review, we identified a total of 32 studies that reported non-tuberculosis mycobacteria (NTM) in Africa. The majority of these studies were conducted in South Africa, followed by Ethiopia and Nigeria. The most commonly isolated NTM species were Mycobacterium avium complex (MAC), Mycobacterium fortuitum, and Mycobacterium abscessus. Many of the studies reported a high prevalence of NTM infections among HIV-positive individuals. Other risk factors for NTM infection included advanced age, chronic lung disease, and previous tuberculosis infection.

**Conclusion:**

In conclusion, this literature review highlights the significant burden of non-tuberculosis mycobacteria infections in Africa. The prevalence of these infections is high, and they are often misdiagnosed due to their similarity to tuberculosis. The lack of awareness and diagnostic tools for non-tuberculosis mycobacteria infections in Africa is a major concern that needs to be addressed urgently. It is crucial to improve laboratory capacity and develop appropriate diagnostic algorithms for these infections.

## Introduction

For decades, the vast and diverse continent of Africa has been grappling with a multitude of infectious diseases that have caused widespread devastation. Among these diseases, tuberculosis (TB) has emerged as a significant public health concern, with staggering morbidity and mortality rates (1,2). The disease has affected millions of people, spreading through crowded urban areas and remote rural communities alike. Despite Mycobacterium tuberculosis (MTB) being the leading cause of TB in Africa, it is important to note that there are other non-tuberculous mycobacteria (NTM) that have also been reported to cause both pulmonary and extrapulmonary infections (3,4).

The prevalence of pulmonary disease caused by non-tuberculous mycobacteria (NTM) is reportedly on the rise worldwide especially in low and middle-income countries with some of the species resistant to various antibiotics (5,6). These non-tuberculosis mycobacteria include Mycobacterium avium complex, Mycobacterium kansasii, Mycobium xenopi, Mycobacterium fortuitum, Mycobacterium abscessus, Mycobacterium marinum and mycobacterium tuberculosis Beijing (3,4,7,8).

The organisms are usually found in the environment, particularly in water and some in the soil (9). These organisms are typically suspected from history and identified through cultures or molecular testing (6,9). However, these non-tuberculous mycobacteria (NTMs) are often misdiagnosed as mycobacterium tuberculosis (MTB) due to their similar clinical presentations, leading to inappropriate treatment and increased morbidity and mortality rates (10,11). The consequences of misdiagnosis can be severe, as NTMs require different treatment regimens and can cause significant damage to the respiratory system if left untreated (12,13). Therefore, it is crucial for clinicians to consider NTMs as a possible diagnosis in patients who do not respond to standard MTB treatment or who have risk factors for NTM infection, such as a compromised immune system.

The main objective of this literature review is to provide a comprehensive overview of the prevalence, clinical manifestations, diagnosis, and management approaches of Non-Tuberculous Mycobacteria (NTM) infections in Africa.

## Methods

To conduct this literature review, a systematic search was performed using various electronic databases including PubMed, Scopus, and Web of Science. The search was limited to studies published in English language from 2000 to 2021. During the literature search, a set of specific keywords were utilized to identify relevant studies related to the prevalence of non-tuberculous mycobacteria (NTM) in Africa. These keywords included “non-tuberculous mycobacteria”, “NTM”, “Africa”, and “prevalence”. Through a rigorous search process, various studies were identified that addressed the prevalence of NTM in different countries in Africa, providing valuable insights into the epidemiology and distribution of NTM infections in this region.

After the initial search, duplicates were removed, and titles and abstracts were screened for relevance. Full-text articles were then reviewed for eligibility based on inclusion criteria which included studies reporting prevalence rates of NTM in African countries. Studies that focused solely on Mycobacterium avium complex (or those that did not report prevalence rates) were excluded.

Data extraction was performed on eligible studies using a standardized form which included information on study design, sample size, geographic location, species of NTM identified, and prevalence rates. Quality assessment of the included studies was also conducted using the Joanna Briggs Institute Critical Appraisal Checklist for Prevalence Studies. Overall, a total of 32 studies met the inclusion criteria and were included in this review.

## Results

In our literature review, we identified a total of 32 studies that reported on non-tuberculous mycobacteria (NTM) in Africa. The majority of these studies were conducted in South Africa, followed by Ethiopia and Nigeria. The most commonly isolated NTM species were Mycobacterium avium complex (MAC), Mycobacterium fortuitum, and Mycobacterium abscessus (13-16). Other species include Mycobacterium chelonae, Mycobacterium kansasii, and Mycobacterium xenopi (16-18).

The studies reported a wide range of prevalence rates ranging from 0.2% to 28%. Interestingly, many of the studies reported a high prevalence of NTM infections among HIV-positive individuals (11, 19,20). MAC was particularly prevalent among this population, with some studies reporting prevalence rates as high as 50% (11,13,19-21). Other risk factors for NTM infection included advanced age, chronic lung disease, and previous tuberculosis infection (19,22).

Common clinical presentations include cough, fever, weight loss, fatigue, chest pain, and shortness of breath. The less common presentations include hemoptysis, pleuritic chest pain, and night sweats (23,24).

The diagnostic tools available in Africa include sputum smear microscopy, chest radiography, and bronchoscopy (19). The availability of these tools varies from country to country and is often limited by cost. The gold standard for diagnosis, which is culture, is not widely available due to the cost and complexity of the procedure. Other diagnostic tests such as PCR and antigen tests are also not widely available due to the cost and complexity of the procedure (19,25). In some cases, clinicians may rely on clinical signs and symptoms to make a diagnosis. In addition, laboratory tests may be used to help confirm a diagnosis. However, these tests can be expensive and time consuming to perform. There is also a major challenge with species identification in most countries. Only few countries are able to do species identification. particularly South Africa, and even then it is not always accurate (19, 25).

The majority of cases of NTM in Africa are treated empirically with a combination of antibiotics. This combination includes macrolides, aminoglycosides, fluoroquinolones, and rifampin (22,26,27). However, this approach is not always successful and lead to the emergence of drug-resistant NTM species.

## Discussion

The presence of non-tuberculous mycobacteria (NTM) in Africa has been a topic of concern for researchers and healthcare providers. This review has highlighted the existing evidence of NTM species and infection prevalence in several countries across Africa. Our literature review revealed that NTM infections are prevalent in Africa. The major isolated NTM species were mycobacterium avium complex (MAC), Mycobacterium fortuitum, and Mycobacterrium abscessus, although other species such as Mycobacterium haemophilum, Mycobacterium xenopi, and Mycobacterium tuberculosis were also identified in many African countries.(14,28,29). The implication of this is that, NTM infections contribute to the burden of respiratory infections leading to morbidity and mortality, and preventive measures should be taken to reduce the spread of these NTM species in the African region. This could be done by the implementation of public health policies to reduce the transmission of these diseases.

Our study also revealed that higher NTM infections incidence in Africa particularly MAC were reported in immune-compromised individuals such as those living with HIV/AIDS. Other risk factors identified were older adults and those with comorbidities or history of tuberculosis infection (30,31). This suggests that attention should be given to individuals of increased vulnerability to developing an NTM infection. This could include increased awareness of the risk factors associated with NTM infections and access to early diagnosis once there is suspicion of the disease for timely treatment (13, 20,32,33).

This study identified lack of awareness and limited access to specific diagnostic tools as the major challenges faced in diagnosing NTM infections (34). Our findings have also revealed that there is disproportionate burden of these infections in this region, with limited resources to diagnose and treat them properly. This leads to misdiagnosis and inappropriate treatment, thereby resulting in prolonged illness and increased healthcare costs. There is also a problem of delayed diagnosis through using solid culture media which is more available in Africa (35-38). The wide availability of liquid culture media and the development of improved RDTs tailored towards specific species will help to improve accuracy and decrease treatment delays caused by misdiagnosis (38). It has also become clear that there is a pressing need for increased awareness and surveillance of NTM infections in Africa. This highlights the urgency of addressing this issue and developing effective strategies to combat NTM infections in Africa.

The limitations of the study include the lack of data on NTM infections in certain African countries, as well as the limited number of studies that have been conducted on NTM infections in Africa. Therefore, increased surveillance is needed to monitor the epidemiology of NTM strains across Africa.

In conclusion, this literature review highlights the significance of recognizing and addressing the growing threat of non-tuberculous mycobacteria infections in Africa to improve patient outcomes and reduce the burden on healthcare systems. The prevalence of these infections is high, and they are often misdiagnosed due to their similarity to tuberculosis. The lack of awareness and diagnostic tools for non-tuberculous mycobacteria infections in Africa is a major concern that needs to be addressed urgently. It is crucial to improve laboratory capacity and develop appropriate diagnostic algorithms for these infections. Additionally, there is a need for increased research on the epidemiology, clinical presentation, and management of non-tuberculosis mycobacteria infections in Africa.
